# Variation in Amygdalin Content in Kernels of Six Almond Species (*Prunus* spp. L.) Distributed in China

**DOI:** 10.3389/fpls.2021.753151

**Published:** 2022-01-28

**Authors:** Wei Wang, Xun-Ze Xiao, Xin-Qiao Xu, Zhen-Jian Li, Jun-Ming Zhang

**Affiliations:** ^1^Key Laboratory of Silviculture of the State Forestry Administration, The Institute of Forestry, The Chinese Academy of Forestry, Beijing, China; ^2^School of Life Sciences, Yulin University, Yulin, China; ^3^Department of Biology, Taiyuan Normal University, Taiyuan, China

**Keywords:** almond species, almond kernel, amygdalin, amygdalin diversity, amygdalin synthesis, environmental factor

## Abstract

Amygdalin, a naturally occurring compound, is one of the main active ingredients of the Chinese raw bitter almond. The variation in amygdalin composition of seed kernels among the six almond species was determined, and relationships with geoenvironmental factors were analyzed. The amygdalin content exhibited great diversity, ranging from 0.0004 to 9.73 g/100 g. The highest level of amygdalin was detected in Tangut almond, with 5.45–9.73 g/100 g. The other kernels showed a range from 3.14 to 6.80 g/100 g in wild almond and from 3.00 to 4.22 g/100 g in longstalk almond. Amygdalin in common almond was almost undetectable. Factor analysis showed that amygdalin content in *Prunus* spp. kernels increased with altitude and decreased with the degree of aspect. Many environmental factors were closely related to amygdalin content, including annual precipitation (Bio12), UV intensity, and topsoil base saturation (T_BS), which all had a significant effect on amygdalin content. The amygdalin content is closely related to rainfall indicators, especially annual precipitation (Bio12), with the highest factor analysis value (3.63). Water regulates amygdalin in diverse ways. Since amygdalin is water-soluble, water can reduce the inhibitory effect of amygdalin on germination and regulate the synthesis of amygdalin at the late stage of germination by activating the amygdalin synthesis genes *CYP79D16* and *CYP71AN24*. This study expands the understanding of amygdalin in almond resources and provides the direction for the regulation of amygdalin.

## Introduction

Amygdalin (D-mandelonitrile-ß-D-gentiobioside), one of the main components of the kernel of Chinese almond fruit, has antioxidative, antibacterial, anti-inflammatory, and immunoregulatory activities (Shi et al., 2019). Cyanogenic glucoside amygdalin initially isolated from the seeds of bitter almonds (*Prunus dulcis* var. amara) is a major component of the kernel of *Rosaceae* plants. For a long time, the potential value of wild almond has been underestimated due to its toxic component, cyanogenic gluoside amygdalin, which affects its development and utilization. However, with the discovery of the nutritional value of wild almond and the potential for amygdalin to prevent and treat conditions such as migraine, hypertension, chronic inflammation, and other diseases, the content change rule of amygdalin and other nutrients or functional components, especially in response to the changing characteristics of environmental factors, has attracted increasing attention (Ayaz et al., 2020).

Almond can be divided into bitter, non-bitter, and sweet species. There are six almond species in China, and the common almond [*Prunus dulcis* (Mill.) D.A.Webb.] is the only species that is mainly distributed in southern Xinjiang Province. Moreover, there are five wild species including high-latitude species such as the wild almond (*Prunus tenella* Batsch), the desert-grown longstalk almond [*Prunus pedunculata* (Pall.) Maxim.], the south-to-north adapted flowering almond (*Prunus triloba* Lindl.), the high-elevation Tangut almond (*Prunus tangutica* (Batalin), and the northern mountainous-soil-grown Mongolic almond (*Prunus mongolica* Maxim.). These wild species of almonds are rich in amygdalin, providing a source for the precious material (Wang et al., 2018, 2019, 2020).

Ecogeographic factors are closely related to plant metabolism (Sampaio et al., 2016) and affect many metabolic pathways in almond, such as fatty acid composition (Nanos et al., 2002; Giovino et al., 2015) and tocopherol concentration (Kodad et al., 2011). Some correlations between ecogeographic factors (temperatures, precipitation, latitude, etc.) and cyanogenic glucoside have been illustrated in plants such as *Trifolium repens* (Majumdar et al., 2004). The amygdalin content varies widely in the *Rosaceae* family, which includes wild almond species. The difference is related to germplasm resources and environmental factors. However, the relationship between environmental factors and amygdalin remains unrevealed in almonds.

Amygdalin synthesis is closely related to the cytochrome P450 family and glycosyltransferase UGT family. The cytochrome P450 (CYP) enzymes *PdCYP79D16* and *PdCYP71AN24* catalyze the conversion of phenylalanine to mandelonitrile, *PdUGT94AF3* is an additional monoglucosyl transferase (UGT) catalyzing prunasin formation, and *PdUGT94AF1* and *PdUGT94AF2* are the two enzymes catalyzing amygdalin formation from prunasin (Thodberg et al., 2018). The transcription of *PdCYP79D16* and *PdCYP71AN24* is controlled by the basic helix-loop-helix transcription factor (*bHLH2*). A non-synonymous point mutation (Leu to Phe) in the dimerization domain of *bHLH2* prevents transcription of the two cytochrome P450 genes, resulting in the sweet kernel trait (Sánchez-Pérez et al., 2019).

In this study, the amygdalin content of ripe almond fruits from 29 regions (including both cultivated and wild varieties) in China was determined. The difference in amygdalin content in kernels was analyzed and evaluated. The main purpose of this study was to clarify the relationship among environmental parameters, genetic variation, and amygdalin content in almond germplasm resources. The results of this study will have a positive impact on the edible and medicinal value of wild almond resources and help to develop new varieties rich in amygdalin.

## Materials and Methods

### Plant Materials

**FIGURE 1:**
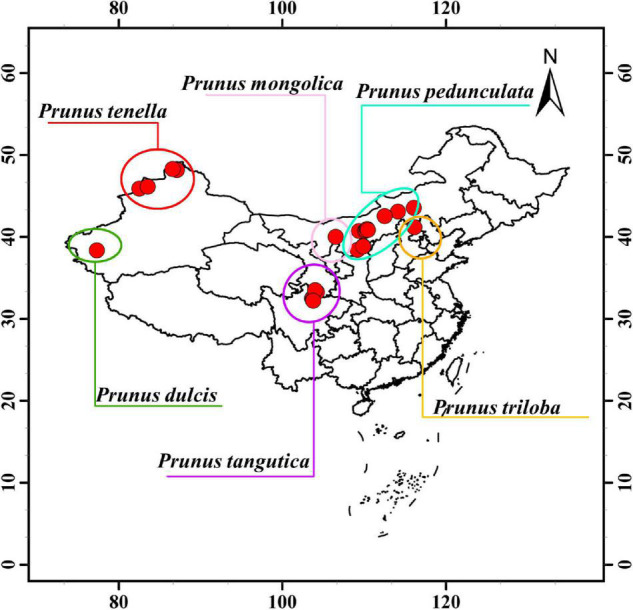
Geographic distribution of different sampled positions of *Prunus* spp. L.

From June to August 2018, kernels from six almond species were obtained from habitats in China according to different maturity periods (Figure 1); including from ten regions in Shaanxi Province and Inner Mongolia Province (longstalk almond), one region in Otog Banner of Inner Mongolia Province (Mongolic almond), seven regions in Aba Prefecture, Sichuan Province (Tangut almond), six regions of Kashi region, Xinjiang Province (common almond), four regions in Altay region of Xinjiang Province (wild almond), and one region in Chengde City of Hebei Province (flowering almond) (Supplementary Table 1). Samples were collected from trees over 20 years old in each region. The degree of maturity was determined by the difficulty of peeling off the green skin and fruit stalk. All samples were dried in the shade and cryopreserved at -20°C until the experiment.

### Extraction and Measurement of Amygdalin

Prior to chemical analyses, the nuts were shelled manually and screened to remove bad seeds. All kernel samples were triturated in liquid nitrogen. The extraction of amygdalin used methanol (20 ml) as the solute, as well as 0.2–0.4 g of almond powder under an ultrasonic generator (500 W, 40 kHz) for 30 min. The supernatant was filtered through filter paper. Then, 1 ml was diluted with an equal volume of 20% methanol and filtered through a 0.22-μm microfiltration membrane for clarification.

High-performance liquid chromatography (HPLC) assays were carried out on a Waters 2650, 2487 UV detector, and a 4.6 × 250 mm C18 column (Waters Sunfire, MA, United States). The mobile phase used was CH_3_OH:H_2_O (20:80, v/v) under isocratic conditions at a flow rate of 1.0 ml/min. A 10-μl sample was injected for analysis. Detection was recorded at 210 nm. The amygdalin used as a standard was purchased from Sigma-Aldrich (MO, United States). The method produced a good separation chromatogram with excellent linearity (correlation *r*^2^ = 0.9999, Supplementary Figure 1) between the peak area and the concentration of amygdalin.

### Environmental Parameters

Geographical data, including longitude, latitude, and altitude, were gathered from habitats using GPS (GARMIN GPSMAP 63SC). Based on the digital elevation model (DEM) derived from SRTM 90M (Shuttle Radar Topographic Mission) data, topographic data, including slope (Slope) and aspect (Aspect), were extracted using ArcGIS 10.2 (version 10.2, ESRI, Redlands, CA, United States). Climate factor data, including 19 factors, such as the annual mean temperature (BIO1), temperature seasonality (BIO4), max temperature of warmest month (BIO5), mean temperature of wettest quarter (BIO8), mean temperature of driest quarter (BIO9), annual precipitation (BIO12), precipitation of wettest month (BIO13), and precipitation seasonality (BIO15) (Bio1-Bio19, more specific information can be found in Table 1), annual mean solar radiation (A_Srad), annual mean water vapor pressure (A_Vapr), and annual mean wind speed (A_Wind) were gathered from the world climate database^1^ using ArcGIS 10.2 with accurate locations to export climate data. Fifteen soil variables (T_BS, T_ESP, T_OC, T_PH_H2O, T_SAND, T_SILT, etc.) were from the Harmonized World Soil Database version 1.2^2^ and included 30 arc-second resolution rasters for topsoil organic carbon, pH, percent silt, and percent sand (Fischer et al., 2008). Global UV-B radiation data (UV-B 1-6) were from the gIUV database^3^ (Manceur et al., 2014). The above factors are shown in Table 1. In this study, forty-eight variables related to the distribution of six almond species were selected, as shown in Supplementary Table 1.

**TABLE 1:** Statistical analysis of amygdalin and environmental factors.

Field	Description	Units	Range	Minimum	Maximum	Mean	SE
Amygdalin	Amygdalin	%	9.73	0.0004	9.73	4.87	0.19
Altitude	Altitude	m	1899.00	900.00	2799.00	1960.37	46.42
Aspect	Aspect	Degree	349.47	0.40	349.87	187.78	7.54
Slope	Slope	%	78.56	0.97	79.53	56.38	2.10
A_Srad	Annual mean solar radiation	kJ/m^2^/day	4325.50	12412.92	16738.42	14392.47	145.45
A_Vapr	Annual mean water vapor pressure	kPa	0.45	0.47	0.92	0.69	0.01
A_Wind	Annual mean wind speed	m/s	2.63	1.38	4.01	2.50	0.06
T_BS	Topsoil base saturation	%	8.00	92.00	100.00	99.79	0.08
T_BULK_DEN	Topsoil reference bulk density	kg/dm^3^	0.30	1.20	1.50	1.38	0.01
T_CACO3	Topsoil calcium carbonate	% weight	9.00	0.00	9.00	6.02	0.19
T_CASO4	Topsoil gypsum	% weight	0.10	0.00	0.10	0.00	0.00
T_CEC_CLAY	Topsoil CEC (clay)	cmol/kg	34.00	46.00	80.00	58.55	0.91
T_CEC_SOIL	Topsoil CEC (soil)	cmol/kg	26.00	1.00	27.00	16.13	0.57
T_CLAY	Topsoil clay fraction	% wt.	22.00	4.00	26.00	17.74	0.49
T_ECE	Topsoil salinity (Elco)	dS/m	0.60	0.10	0.70	0.27	0.01
T_ESP	Topsoil sodicity (ESP)	%	6.00	0.00	6.00	1.85	0.10
T_GRAVEL	Topsoil Gravel Content	%vol.	13.00	3.00	16.00	8.16	0.33
T_OC	Topsoil organic carbon	% weight	2.01	0.40	2.41	0.97	0.05
T_PH_H2O	Topsoil pH (H2O)		2.70	5.70	8.40	7.75	0.05
T_REF_BULK	Topsoil reference bulk density	kg/dm^3^	0.39	1.35	1.74	1.45	0.01
T_SAND	Topsoil Sand Fraction	% wt.	66.00	24.00	90.00	45.90	1.75
T_SILT	Topsoil silt fraction	% wt.	48.00	6.00	54.00	36.36	1.28
Bio1	Annual mean temperature	°C	11.10	1.45	12.55	6.12	0.19
Bio2	Mean diurnal range [mean of monthly (max temp–min temp)]	°C	4.03	10.34	14.38	12.07	0.11
Bio3	Isothermality (BIO2/BIO7) (* 100)	°C	13.63	23.53	37.16	32.31	0.34
Bio4	Temperature seasonality (standard deviation *100)	%	766.24	662.27	1428.51	947.83	22.78
Bio5	Max temperature of warmest month	°C	13.00	19.80	32.80	24.19	0.27
Bio6	Min temperature of coldest month	°C	18.30	-24.80	-6.50	-14.12	0.48
Bio7	Temperature annual range (BIO5-BIO6)	°C	20.70	29.90	50.60	38.31	0.68
Bio8	Mean temperature of wettest quarter	°C	12.78	12.07	24.85	15.93	0.21
Bio9	Mean temperature of driest quarter	°C	22.10	-17.12	4.98	-5.86	0.40
Bio10	Mean temperature of warmest quarter	°C	11.30	13.55	24.85	17.24	0.22
Bio11	Mean temperature of coldest quarter	°C	17.33	-17.12	0.22	-6.22	0.43
Bio12	Annual precipitation	mm	689.00	38.00	727.00	513.33	15.71
Bio13	Precipitation of wettest month	mm	121.00	8.00	129.00	102.83	1.62
Bio14	Precipitation of driest month	mm	10.00	0.00	10.00	2.71	0.12
Bio15	Precipitation seasonality (coefficient of variation)	%	71.93	40.26	112.19	91.03	1.38
Bio16	Precipitation of wettest quarter	mm	324.00	17.00	341.00	270.45	5.63
Bio17	Precipitation of driest quarter	mm	31.00	2.00	33.00	12.48	0.37
Bio18	Precipitation of warmest quarter	mm	324.00	17.00	341.00	263.97	5.55
Bio19	Precipitation of coldest quarter	mm	40.00	3.00	43.00	12.61	0.42
UVB1	Annual mean UV-B	J/m^2^/day	2685.33	2204.83	4890.16	3530.66	75.34
UVB2	UV-B seasonality	J/m^2^/day	118944.00	114355.00	233299.00	182413.92	2841.44
UVB3	Mean UV-B of highest_month	J/m^2^/day	3433.31	4363.33	7796.64	5875.74	101.23
UVB4	Mean UV-B of lowest month	J/m^2^/day	1426.32	296.98	1723.30	1126.55	40.99
UVB5	UV-B radiation of highest quarter	J/m^2^/day	10102.50	12047.50	22150.00	16519.49	301.38
UVB6	Sum of UV-B radiation of lowest quarter	J/m^2^/day	5867.20	2015.48	7882.68	5351.33	153.20

### Water Treatment

The seeds of longstalk almonds were soaked in twice their volume of pure water for 7 days, and the water was changed every day. Then, the soaked seeds were treated with sterilized river sand for 9 days at 25°C. Some shelled kernels (2.0 g) were randomly selected to test the moisture content after each sampling. The rest of the kernels were ground in a mortar under liquid nitrogen. Then, the crushed samples were wrapped with aluminum foil paper and stored at -80°C.

### Moisture Detection of Shelled Kernel

The seed kernel samples were weighed in a glass dish and then, dried in an oven at 105°C for 1 h. After taking the samples out, the dry weight was measured. Each sample was measured three times, and the average value was obtained.

### RNA Isolation and Gene Expression Analysis

The primers used in this study refer to six amygdalin synthesis gene primers reported by Thodberg et al. (2018). The primers were synthesized by Shenggong Bioengineering (Shanghai) Co., Ltd. *UBQ10* was the internal reference gene. All primers were purified by HPLC (Supplementary Table 2).

For RNA extraction, 0.2 g of crushed samples were collected from the corresponding plants, and RNAprep pure plant kits were used in strict accordance with the requirements of the manufacturer. The determination of RNA content and quality was mainly performed using a NanoDrop 2000 (Thermo Fisher Scientific, Waltham, MA, United States). cDNA was synthesized by using a reverse transcription kit according to the instructions (PrimeScript™RT Master Mix, TaKaRa Biotechnology Co., Ltd. Dalian, China). RT-PCR experiments were conducted using previously reported methods (real-time quantitative PCR was performed using the KAPA SYBR FAST qPCR Master Mix kit). PCRs were carried out using high-fidelity Phusion DNA polymerase. The primer sequences of the test genes were consistent with previous studies (Thodberg et al., 2018). The cycle threshold (CT) 2^–ΔΔCT^ method was used to measure the relative expression of the tested gene. All the samples were analyzed in triplicate.

Parameter settings for real-time fluorescence quantitative PCR are as follows:

1. Predenaturation: temperature 95°C, retention time 30 s (temperature rising and falling speed: 4.4°C/s), 1 cycle;
2. Amplification: temperature 95°C, retention time 5 s (temperature rise and fall rate: 4.4°C/s), temperature 60°C, retention time 30 s (temperature rise and fall speed: 2.2°C/s), 40 cycles;
3. Melting: temperature 95°C, retention time 5 s (temperature rise and fall speed: 4.4°C/s), temperature 60°C, retention time 1 min (temperature rise and fall speed: 2.2°C/s), temperature 95°C (temperature rise and fall speed: 4.4°C/s), 1 cycle;
4. Cooling: temperature 50°C (temperature rise and fall speed: 2.2°C/s), 1 cycle.

Data processing:

The CT value data were analyzed by relative quantitative analysis (*UBQ10* as the internal reference gene) and sorted into 2^–ΔΔ*CT*^ mean values, which were positively correlated with the expression level, as shown in Formula (1).


(1) Relative⁢expression⁢of⁢the⁢target⁢gene⁢in⁢each⁢sample=2(mean)-ΔΔC⁢T

Δ⁢Δ⁢CT=Δ⁢CT-Δ⁢CT⁢(mean)

ΔCT=CT(target)-CT(ref.mean)

Symbols of formulas:

CT (target)—Cycle number of the target gene in the sample;

CT (ref.mean)—The average number of internal reference gene cycles in three replicates;

ΔCT (mean)—Average ΔCT values of three repeats of the target gene in the sample.

### Statistical Analyses

Interspecific and intraspecific data on amygdalin content and environmental factors were integrated. The Pearson correlation analysis between amygdalin and other environmental data and the CABFAC factor analysis of environmental factors on amygdalin content were carried out using Paleontological Statistics (PAST) software program Version 3.14 (Hammer et al., 2001). Figures were drawn using Sigmaplot 14 (version 14, Systat Software Inc., Chicago, IL, United States) and MATLAB R2014a (version R2014a, MathWorks, Natick, MA, United States), and tables were created in Excel 2010.

## Results and Discussion

### Sample Collection

Wild almond species are widely distributed in the world showing a large span in both latitude and longitude. In this study, 145 wild almond germplasm resources were collected in the natural distribution area of wild almond, and 6 almond cultivars were collected in the main cultivation area of almond (Supplementary Table 1). The span of the sample collection area covers the natural distribution areas of six almond species in mainland China, spanning the longitude range of 38°46′12″ and the latitude range of 16°2′33″ (Figure 1), with an altitude span of 1,960.37 m (Figure 1 and Table 1).

### Variation of Amygdalin Content in Almond Germplasm

**FIGURE 2:**
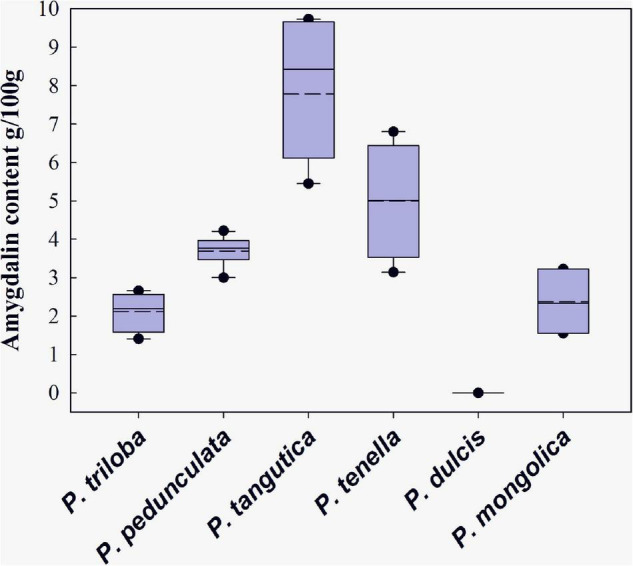
Range of amygdalin contents in six almond species in China. Medium dashed lines show each mean value, and solid lines show each median.

The variation range of amygdalin contents in six species seeds is given in Figure 2. Among the species analyzed, amygdalin was most abundant in Tangut almond, with the highest amygdalin content (9.73 g/100 g) and the highest average (7.78 g/100 g). The amygdalin content in wild almond was highly related to the germplasm, with an average of 3.14–6.80 g/100 g. Longstalk almond was the most sampled, and its amygdalin content varied from 3.00 to 4.22 g/100 g. The contents of flowering almond and Mongolic almond seeds were slightly lower at 1.41–2.65 and 1.55–3.22 g/100 g, respectively. Nearly no amygdalin was found in common almond. Figure 2 shows that the amygdalin content of Tangut almond and wild almond fluctuates greatly. In addition, the median amount of amygdalin in Tangut almond (8.47 g/100 g) was much higher than the average, while the median amount of amygdalin in other almond resources was close to the average. These results may stem from genotype adaptation to the environment.

Among the other species studied, Tangut almond and wild almond represent the highest levels of amygdalin, which are extremely high compared with other species in *Rosaceae* as well. Amygdalin contents similar to Tangut almond have been reported in the bitter apricot (*Prunus armeniaca* L.) cultivars “Palstein,” “Canino,” and “Tirynthos” (Gómez et al., 1998), and it was also reported that some Turkish bitter apricot species (“Paviot,” “Karacabey,” and “Alyanak”) (Yildirim and Askin, 2010) have approximately the same content as wild almond. Longstalk almond represents the middle level of amygdalin among the six species, with a range from 3.00 to 4.22 g/100 g (Lee et al., 2013). Mongolic almond and flowering almond are considered to have low levels, which is similar to the slightly bitter almond with a reported concentration of 2.25 g/100 g (Yildirim and Askin, 2010). According to the Chinese pharmacopeia, the amygdalin content of almond kernels should be more than 3.0 g/100 g. Therefore, longstalk almond, Tangut almond, and wild almond are suitable as raw materials for traditional Chinese medicine (Chinese-pharmacopoeia-commission, 2015).

Amygdalin content has a significant variation among almond species. Cyanide such as amygdalin concentration in plants is highly related to environmental factors (Pederson et al., 1996; Niedźwiedź-Siegień and Gierasimiuk, 2001). Considering the disparate amygdalin contents and habitat range among different almond species, it is plausible to consider that location and climate factors have an effect on amygdalin content.

### Contribution of Ecogeographic Variables to Amygdalin Contents

**FIGURE 3:**
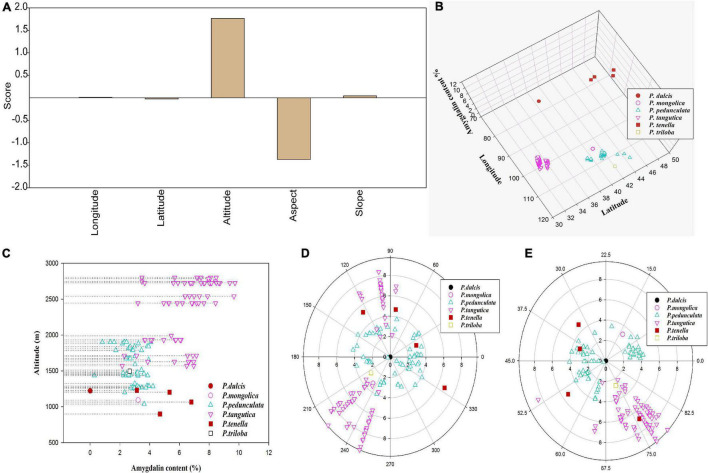
Factor analysis scores of amygdalin content in five topographic datasets (longitude, latitude, altitude, slope, and aspect) of six almond species **(A)**; amygdalin content in different longitudes, latitudes **(B)**, and altitudes **(C)**; scatter plot of amygdalin content in different aspects **(D)** and slopes **(E)**.

Although the exact reasons for the high amygdalin content in the kernels of wild almond have not been elucidated, it has been speculated that the unique geographical environments (longitude and latitude, altitude, aspect, slope, and corresponding climate change) and genomic differences could greatly influence the biosynthesis and accumulation of amygdalin. Therefore, this study first explores the correlation between amygdalin content and ecogeographic variables (Figure 3).

Factor analysis showed that amygdalin content in *Prunus* spp. kernels increased with altitude (Figures 3A,C and Supplementary Table 3), decreased with the degree of aspect (Figures 3A,D and Supplementary Table 3), and had less relationship with longitude, latitude (Figures 3A,B and Supplementary Table 3), and slope (Figures 3A,E and Supplementary Table 3). The elevation of altitude is often accompanied by an increase in UV intensity, a decrease in temperature, and an increase in illumination hours. These changes in the external environment may be the main reason for the increase in secondary metabolites including amygdalin (Liu et al., 2020). With increasing altitude, the amygdalin content increases, so this may be one of the characteristics of almonds adapting to the harsh features of high-altitude climates, including high UV radiation. As shown in Figure 3D, the distribution of almond resources is mostly concentrated on sunny and partial sunny slopes with an aspect of 90–270°, indicating that almond resources are photophilous plants. The correlation between geographical factors (such as altitude, slope, and aspect) and amygdalin may be affected by the difference in light and UV light.

### Contribution of Climate Variables to Amygdalin Contents

Different regions of almond habitats bring diverse environmental factors, mainly changing precipitation and temperature. In this study, common almond [*Prunus dulcis* (Mill.) D.A.Webb.] is the only species mainly distributed in southern Xinjiang Province; wild almond (*Prunus tenella* Batsch) is distributed in the high latitudes of northern Xinjiang Province; and longstalk almond [*Prunus pedunculata* (Pall.) Maxim.] is distributed in deserts and rocky mountainous areas such as Shaanxi and Inner Mongolia. In addition, there are south-to-north-adapted flowering almond (*Prunus triloba* Lindl.), high-elevation Tangut almond (*Prunus tangutica* (Batalin), and northern mountainous-soil-grown Mongolic almond (*Prunus mongolica* Maxim.) (Figure 1). Among the sample collection regions, total annual precipitation ranges from 38 to 727 mm and the annual average temperature ranges from 1.45 to 12.55°C. The highest precipitation was found in Tangut almond habitat, which receives 610–727 mm. The second was the flowering almond habitat with about 476 mm. Precipitation of longstalk almond, Mongolic almond, and wild almond habitats was between 100 and 400 mm. The lowest was observed in the common almond habitat, with 38–85 mm. Conversely, the annual average temperature in the common almond habitat was the highest: about 12.55°C. The habitats of other species show the same temperature span of 1.45–10.02°C (Table 1).

**FIGURE 4:**
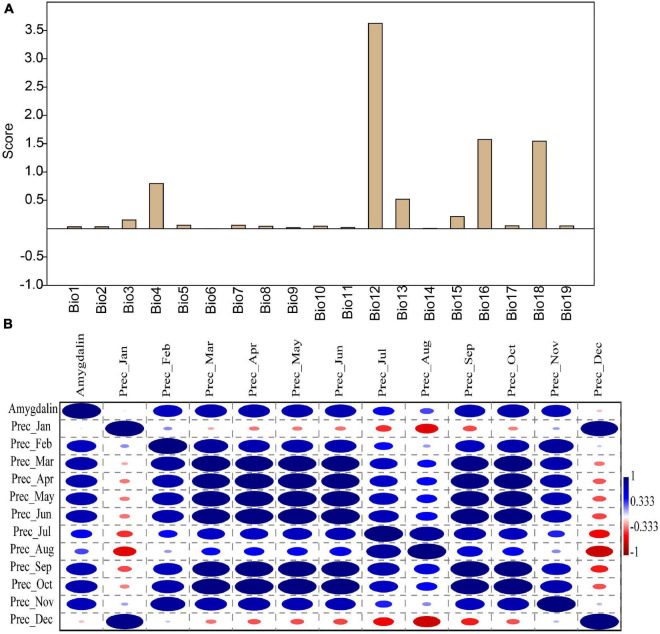
Factor analysis scores of amygdalin content in 19 climate factor datasets (Bio1–Bio19) of six almond varieties **(A)**. Correlation analysis between amygdalin content and precipitation from January to December **(B)**. The correlation coefficients ranged from 1 (blue, positive) to -1 (red, negative).

The factor analysis function of PAST3 software was used to analyze the contribution of 19 temperature and rainfall-related data to amygdalin content. The results showed that among the temperature-related factors (Bio1–Bio11), only the factor analysis score of temperature seasonality (Bio4) exceeded 0.5 (Figures 4A,B and Supplementary Table 4). Other temperature indexes had less effect on amygdalin content. However, amygdalin content was closely related to rainfall indicators, especially annual precipitation (Bio12). The factor analysis value was the highest for annual precipitation (3.63), followed by 1.57 and 1.54 for the precipitation of the wettest quarter (Bio16) and the precipitation of the warmest quarter (Bio18). Both Bio16 and Bio18 contributed more than 1.5 to the amygdalin content (Figure 4A). The chemical composition of plant seeds is closely related to rainfall, irrigation, and drought. Tangolar et al. (2019) showed that the phytochemical contents of grape seeds changed according to irrigation levels. There was a significant negative correlation between soybean seed oil and rainfall (Solanki et al., 2007). Rainfall may further affect the chemical composition of plant seeds by changing the water content in the soil and atmosphere. However, how the amygdalin content changes in almond kernels corresponding to rainfall needs further study.

### Correlation Between Amygdalin Contents and Other Environmental Indicators

**FIGURE 5:**
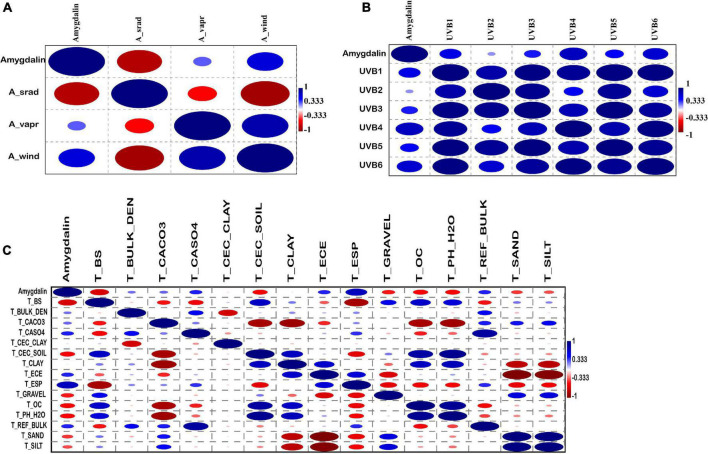
Correlation coefficient between amygdalin content and 3 annual climate factor datasets (A_Srad, annual mean solar radiation; A_Vapr, annual mean water vapor pressure; A_Wind, annual mean wind speed). **(A)** Correlation coefficient between amygdalin content and six UV-B radiation datasets. **(B)** Correlation coefficient between 15 soil variables and amygdalin content. **(C)** The correlation coefficients ranged from 1 (blue, positive) to -1 (red, negative).

The correlation between amygdalin and other environmental data was also analyzed using PAST3 software. The results showed that amygdalin was positively correlated with the annual mean wind speed (A_Wind), was negatively correlated with the annual mean solar radiation (A_Srad), and had a low correlation coefficient with the annual mean water vapor pressure (A_Vapr) (Figure 5A and Supplementary Table 5). Almond species are light-preferring plants, but an increase in light radiation beyond a certain range is not conducive to the synthesis of amygdalin, while an increase in wind speed can promote amygdalin synthesis. As shown in Figure 5B and Supplementary Table 6, the amygdalin content was positively correlated with the six UV intensity indices, which indicated that the amygdalin content was closely related to the intensity of UV radiation. UV radiation is a small fraction of the solar spectrum which acts as a key environmental modulator of plant function, affecting metabolic regulation and growth (Wang et al., 2010; Reyes et al., 2020). Therefore, the increase of amygdalin content with elevation may be due to the change of UV intensity. The highest positive correlation coefficient was of amygdalin and the topsoil sodicity (T_ESP) with 0.75, while the lowest negative coefficient was the topsoil base saturation (T_BS) with -0.65 (Supplementary Table 7). The correlation coefficients of other soil indexes were generally low (Figure 5C and Supplementary Table 7). There was a positive correlation between the topsoil sodicity and amygdalin, indicating that increased soil salinity was beneficial to amygdalin synthesis.

### Effect of Water on Amygdalin Content of the Wild Almond Kernel

**FIGURE 6:**
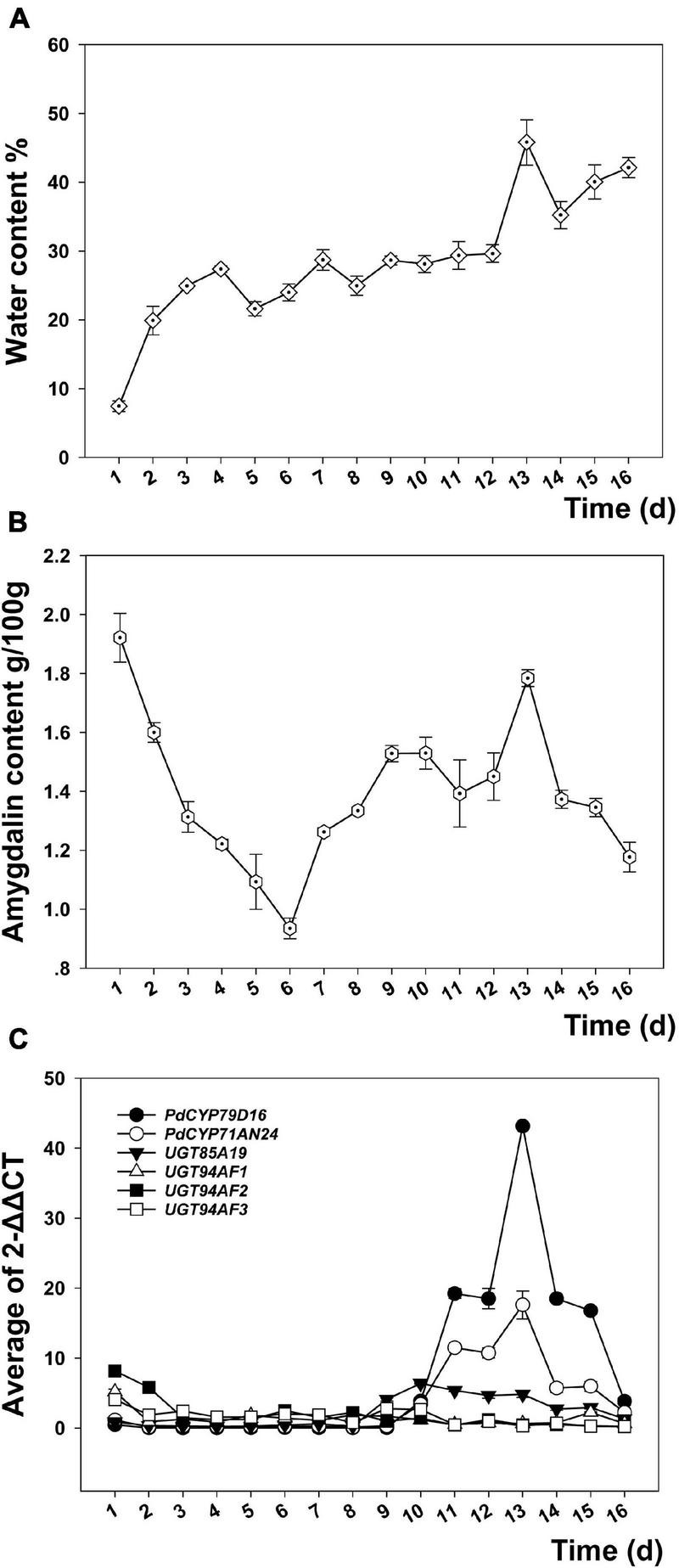
Changes in the water content **(A)**, amygdalin content **(B)**, and expression of key regulatory genes **(C)** in kernels of longstalk almond during the process of seed germination promoted by water treatment.

The indices related to water and the factors affecting water, such as the annual precipitation (Bio12) and the annual mean solar radiation (A_Srad), have positive effects on amygdalin content. Therefore, we studied the effect of water treatment on amygdalin content in kernels. During the seed germination promoted by water immersion, the kernel water content continued to increase. There was a small peak on the 7th day and the largest peak on the 13th day (Figure 6A). The amygdalin content decreased in the early stage, but increased after the 6th day and reached a peak on the 13th day (Figure 6B). The P450 gene families *CYP79D16* and *CYP71AN24* showed similar expression patterns. The expression level was not high before the 10th day, with only a small peak appearing on the 7th day. A larger peak appeared on the 11th day while the highest peak occurred on the 13th day, and then expression decreased to the previous level by the 16th day (Figure 6C). In contrast, the expression peaks of four glycosyltransferase genes (i.e., *UGT85A19*, *UGT94AF1*, *UGT94AF2*, and *UGT94AF3*) appeared in the fruit development stage (Thodberg et al., 2018). The expression levels of these four genes did not fluctuate significantly during seed germination.

Amygdalin has the effect of inhibiting seed germination, providing a nitrogen source for plant growth and insect resistance, which can be coordinated by water. In the early stage of water treatment, water can dissolve amygdalin to reduce the amount of amygdalin in the seed kernel and relieve its inhibition on seed germination to a certain extent. After seed germination, amygdalin synthesis is initiated by two key enzymes, namely, *CYP79D16* and *CYP71AN24*, which regulate amygdalin synthesis (Thodberg et al., 2018) to avoid insect pests in the early stage of seed germination. The response of amygdalin content to water is favorable to avoid seed germination in periods of drought. Almond germplasm may reduce its demand for precipitation in arid areas by regulating the amygdalin content.

## Conclusion

In this study, a large-scale study and evaluation of amygdalin content in wild and cultivated almond kernels were carried out for the first time. Almond resources contain abundant amygdalin, which indicates that kernels are an excellent source of amygdalin. The results also showed that different almond germplasm resources have great variation in kernel amygdalin content. Longstalk almond, Tangut almond, and wild almond are suitable as raw materials for traditional Chinese medicine.

This study also points out that some environmental factors were closely related to amygdalin content, among which altitude, aspect, annual precipitation (Bio12), UV intensity, topsoil sodicity (T_ESP), and topsoil base saturation (T_BS) significantly affected the amygdalin content. This provides a direction for future research on amygdalin regulation. It also provides a method for reference for the relationship between other plant metabolites and environmental factors.

During the germination of almond seeds, water had a synergistic effect on seed germination ability by regulating the amygdalin content. At the early stages of germination, water decreased the inhibitory effect of amygdalin on germination. At the later stage, amygdalin was synthesized by regulating key genes to improve the insect resistance of the plants. Almonds may utilize the water-soluble nature of amygdalin and activate amygdalin synthesis genes for the purpose of regulating amygdalin to play an important physiological role in almond seed germination. The correlation between amygdalin concentration and water stress needs further research.

## Data Availability Statement

The original contributions presented in the study are included in the article/Supplementary Material, further inquiries can be directed to the corresponding author/s.

## Author Contributions

WW conceived and X-QX designed the study. WW and X-ZX processed the data, performed the analyses and analyzed the results, and wrote the manuscript. J-MZ and Z-JL edited the manuscript. All authors read and approved the final version of the manuscript.

## Conflict of Interest

The authors declare that the research was conducted in the absence of any commercial or financial relationships that could be construed as a potential conflict of interest.

## Publisher’s Note

All claims expressed in this article are solely those of the authors and do not necessarily represent those of their affiliated organizations, or those of the publisher, the editors and the reviewers. Any product that may be evaluated in this article, or claim that may be made by its manufacturer, is not guaranteed or endorsed by the publisher.

## Footnotes

1
http://www.worldclim.org/

2
http://www.fao.org/soils-portal/soil-survey/soil-maps-and-databases/harmonized-world-soil-database-v12/en/

3
http://www.ufz.de/gluv/

## Supplementary Material

Supplementary Figure 1 Standard curve of HPLC assay in amygdalin detection.
