# Supplementary figures and images for: Variation in Amygdalin Content in Kernels of Six Almond Species (Prunus spp. L.) Distributed in China
Source: Front Plant Sci. 2022 Jan 28;12:753151. doi: 10.3389/fpls.2021.753151 (PMC8831915; doi:10.3389/fpls.2021.753151)

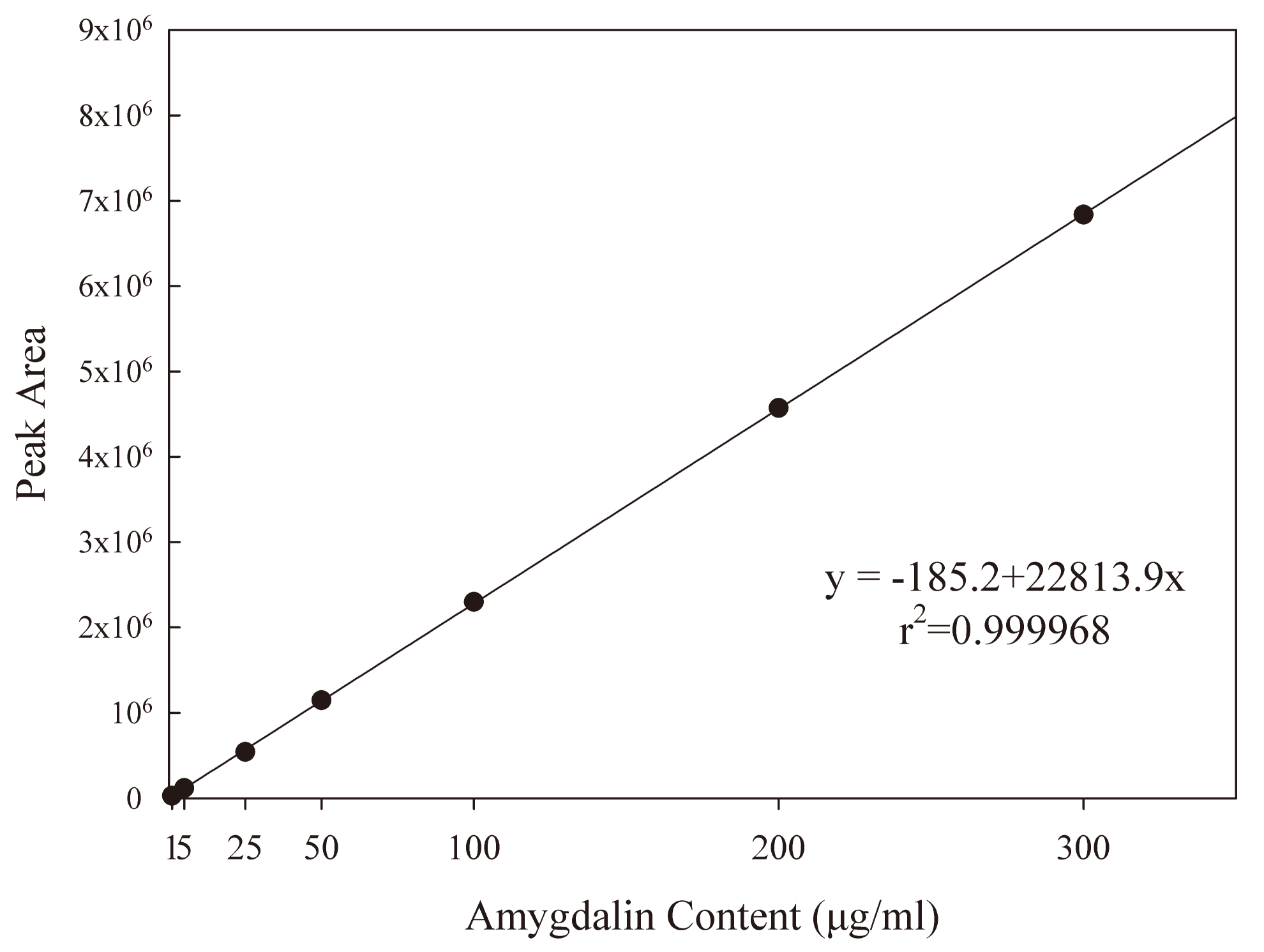

Supplement: Supplementary Figure 1 — Standard curve of HPLC assay in amygdalin detection. [file Image_1.TIF]
