# Supplementary material for: Variation in Amygdalin Content in Kernels of Six Almond Species (Prunus spp. L.) Distributed in China
Source: Front Plant Sci. 2022 Jan 28;12:753151. doi: 10.3389/fpls.2021.753151 (PMC8831915; doi:10.3389/fpls.2021.753151)
Supplement: Supplementary file 2 [file Table_1.DOCX]

Table 1S Sampling details for *Prunus* spp. L. collections.

| Latin Name of Species | Locality | Code | Sample No. |
| --- | --- | --- | --- |
| *P. dulcis* | Kashi, Xinjiang | blm | 6 |
| *P. mongolica* | Otog Banner, Inner Mongolia | mg | 1 |
| *P. pedunculata* | Xilinhot, Inner Mongolia | lks | 1 |
| *P. pedunculata* | Sonid Right Banner, Inner Mongolia | sntyq | 1 |
| *P. pedunculata* | Sunid Left Banner, Inner Mongolia | sz | 1 |
| *P. pedunculata* | Damiao village, Guyang, Inner Mongolia | dmc | 5 |
| *P. pedunculata* | Yuyang , Shaanxi | yy | 5 |
| *P. pedunculata* | Meilinggou, Guyang, Inner Mongolia | mlg | 6 |
| *P. pedunculata* | Bayannaoer, Inner Mongolia | wls | 8 |
| *P. pedunculata* | Saihudong, Guyang, Inner Mongolia | shd | 11 |
| *P. pedunculata* | Shenmu , Shaanxi | sm | 11 |
| *P. pedunculata* | Guanfugou, Guyang, Inner Mongolia | gfg | 12 |
| *P. tangutica* | Tianxugudi, Aba Prefecture, Sichuan | txgd | 1 |
| *P. tangutica* | Gedaba, Aba Prefecture, Sichuan | gdb | 8 |
| *P. tangutica* | Anhong, Aba Prefecture, Sichuan | ah | 11 |
| *P. tangutica* | Toudaocheng, Aba Prefecture, Sichuan | tdc | 11 |
| *P. tangutica* | Palagou, Aba Prefecture, Sichuan | plg | 13 |
| *P. tangutica* | Zhenping, Aba Prefecture, Sichuan | zpx | 14 |
| *P. tangutica* | Deshengbao, Aba Prefecture, Sichuan | dsb | 20 |
| *P. tenella* | Buerjin, Xinjiang | che | 1 |
| *P. tenella* | Guozigou,Tacheng, Xinjiang | gzg | 1 |
| *P. tenella* | Habahe, Xinjiang Xin | ktl | 1 |
| *P. tenella* | Yumin,Tacheng, Xinjiang | ym | 1 |
| *P. triloba* | Chengde, Hebei | bc | 1 |
