# Supplementary material for: Variation in Amygdalin Content in Kernels of Six Almond Species (Prunus spp. L.) Distributed in China
Source: Front Plant Sci. 2022 Jan 28;12:753151. doi: 10.3389/fpls.2021.753151 (PMC8831915; doi:10.3389/fpls.2021.753151)
Supplement: Supplementary file 3 [file Table_2.DOCX]

Table 2S Primer of amygdalin synthesis and reference gene.

| Primer | Sequence(5'-3') | Tm℃ |
| --- | --- | --- |
| *CYP79D16* | TAAGGCCTTGGCCTATTGTG | 60 |
|  | TGTGTATCCACCGGAATGTG | 60 |
| *CYP71AN24* | TTGCAGAGTTGGTCAGCAAG | 60 |
|  | TCTGTCCAAGAATGCACCTG | 60 |
| *UGT85A19* | CACATGCAGTTTGCATACCC | 60 |
|  | AGGCGTTTGTGGTTGAACTC | 60 |
| *UGT94AF1* | CAATGCAGTGCTCGATCTTG | 60 |
|  | TGAACAAGTGTGCCAACAGG | 60 |
| *UGT94AF2* | AATGCAGTGCTCGATCATGC | 60 |
|  | TTGAACAAGCGGACCAACTG | 60 |
| *UGT94AF3* | CCAATTGTAGCCATGCCTATGC | 60 |
|  | TTCTTCCACCACCACATCTCTG | 60 |
| *UBQ10 (*Reference gene*)* | AAGGCTAAGATCCAAGACAAAGAG | 60 |
|  | CCACGAAGACGAAGCACTAAG | 60 |
