# Supplementary material for: Variation in Amygdalin Content in Kernels of Six Almond Species (Prunus spp. L.) Distributed in China
Source: Front Plant Sci. 2022 Jan 28;12:753151. doi: 10.3389/fpls.2021.753151 (PMC8831915; doi:10.3389/fpls.2021.753151)
Supplement: Supplementary file 4 [file Table_3.DOCX]

Table 3S CABFAC factor analysis of five topographic (longitude, latitude, altitude, slope, and aspect) based on the basis of amygdalin content.

|  | Factor 1 | Factor 2 | Factor 3 | Factor 4 |
| --- | --- | --- | --- | --- |
| Longitude | 0.01 | -0.18 | -1.75 | -1.03 |
| Latitude | -0.02 | -0.09 | -0.72 | -0.57 |
| Altitude | 1.76 | -1.37 | 0.07 | 0.12 |
| Aspect | -1.37 | -1.76 | 0.13 | 0.09 |
| Slope | 0.04 | -0.05 | 1.18 | -1.89 |
