# Supplementary material for: Variation in Amygdalin Content in Kernels of Six Almond Species (Prunus spp. L.) Distributed in China
Source: Front Plant Sci. 2022 Jan 28;12:753151. doi: 10.3389/fpls.2021.753151 (PMC8831915; doi:10.3389/fpls.2021.753151)
Supplement: Supplementary file 5 [file Table_4.DOCX]

Table 4S CABFAC factor analysis of 19 climate factor data (Bio1-Bio19) based on amygdalin content.

|  | Factor 1 | Factor 2 | Factor 3 | Factor 4 |
| --- | --- | --- | --- | --- |
| Bio1 | 0.03 | 0.00 | 0.02 | 1.28 |
| Bio2 | 0.03 | 0.03 | 0.02 | 0.09 |
| Bio3 | 0.15 | 0.02 | 0.21 | 0.48 |
| Bio4 | 0.80 | 4.26 | 0.39 | 0.07 |
| Bio5 | 0.06 | 0.07 | 0.06 | 1.33 |
| Bio6 | 0.00 | -0.07 | 0.05 | 1.21 |
| Bio7 | 0.06 | 0.14 | 0.01 | 0.12 |
| Bio8 | 0.04 | 0.05 | 0.03 | 1.39 |
| Bio9 | 0.02 | -0.05 | 0.07 | 1.36 |
| Bio10 | 0.04 | 0.05 | 0.02 | 1.27 |
| Bio11 | 0.02 | -0.05 | 0.02 | 1.26 |
| Bio12 | 3.63 | -0.86 | 2.06 | -0.06 |
| Bio13 | 0.52 | 0.03 | -1.32 | -0.57 |
| Bio14 | 0.00 | 0.01 | 0.13 | -0.54 |
| Bio15 | 0.21 | 0.27 | -1.57 | -0.89 |
| Bio16 | 1.57 | -0.12 | -2.20 | 0.53 |
| Bio17 | 0.05 | 0.02 | 0.52 | -1.60 |
| Bio18 | 1.54 | -0.12 | -2.20 | -0.25 |
| Bio19 | 0.05 | 0.02 | 0.62 | -1.60 |
