# Supplementary material for: Variation in Amygdalin Content in Kernels of Six Almond Species (Prunus spp. L.) Distributed in China
Source: Front Plant Sci. 2022 Jan 28;12:753151. doi: 10.3389/fpls.2021.753151 (PMC8831915; doi:10.3389/fpls.2021.753151)
Supplement: Supplementary file 6 [file Table_5.DOCX]

Table 5S Pearson linear correlation coefficient between amygdalin content and annual climate factors.

|  | Amygdalin | A_srad | A_vapr | A_wind |
| --- | --- | --- | --- | --- |
| Amygdalin |  | -0.80 | 0.32 | 0.64 |
| A_srad | -0.80 |  | -0.51 | -0.86 |
| A_vapr | 0.32 | -0.51 |  | 0.82 |
| A_wind | 0.64 | -0.86 | 0.82 |  |
