# Supplementary material for: Variation in Amygdalin Content in Kernels of Six Almond Species (Prunus spp. L.) Distributed in China
Source: Front Plant Sci. 2022 Jan 28;12:753151. doi: 10.3389/fpls.2021.753151 (PMC8831915; doi:10.3389/fpls.2021.753151)
Supplement: Supplementary file 7 [file Table_6.DOCX]

Table 6S Pearson linear correlation coefficient between amygdalin content and ultraviolet radiation data.

|  | Amygdalin | UVB1 | UVB2 | UVB3 | UVB4 | UVB5 | UVB6 |
| --- | --- | --- | --- | --- | --- | --- | --- |
| Amygdalin |  | 0.59 | 0.22 | 0.45 | 0.75 | 0.51 | 0.69 |
| UVB1 | 0.59 |  | 0.84 | 0.97 | 0.91 | 0.99 | 0.97 |
| UVB2 | 0.22 | 0.84 |  | 0.94 | 0.53 | 0.91 | 0.69 |
| UVB3 | 0.45 | 0.97 | 0.94 |  | 0.79 | 1.00 | 0.89 |
| UVB4 | 0.75 | 0.91 | 0.53 | 0.79 |  | 0.83 | 0.98 |
| UVB5 | 0.51 | 0.99 | 0.91 | 1.00 | 0.83 |  | 0.93 |
| UVB6 | 0.69 | 0.97 | 0.69 | 0.89 | 0.98 | 0.93 |  |
