# Supplementary material for: Variation in Amygdalin Content in Kernels of Six Almond Species (Prunus spp. L.) Distributed in China
Source: Front Plant Sci. 2022 Jan 28;12:753151. doi: 10.3389/fpls.2021.753151 (PMC8831915; doi:10.3389/fpls.2021.753151)
Supplement: Supplementary file 8 [file Table_7.DOCX]

Table 7S Pearson linear correlation coefficient between amygdalin content and fifteen soil variables.

|  | Amygdalin | T_BS | T_BULK_DEN | T_CACO3 | T_CASO4 | T_CEC_CLAY | T_CEC_SOIL | T_CLAY | T_ECE | T_ESP | T_GRAVEL | T_OC | T_PH_H2O | T_REF_BULK | T_SAND | T_SILT |
| --- | --- | --- | --- | --- | --- | --- | --- | --- | --- | --- | --- | --- | --- | --- | --- | --- |
| Amygdalin |  | -0.65 | 0.28 | 0.30 | 0.44 | -0.08 | -0.52 | -0.06 | 0.44 | 0.75 | -0.46 | -0.52 | -0.52 | 0.44 | -0.39 | -0.35 |
| T_BS | -0.65 |  | -0.12 | -0.47 | -0.53 | 0.11 | 0.73 | 0.27 | -0.33 | -0.84 | 0.56 | 0.71 | 0.65 | -0.47 | 0.27 | 0.24 |
| T_BULK_DEN | 0.28 | -0.12 |  | -0.03 | 0.53 | -0.67 | 0.11 | 0.23 | 0.14 | 0.24 | 0.07 | -0.10 | -0.11 | 0.50 | -0.15 | -0.09 |
| T_CACO3 | 0.30 | -0.47 | -0.03 |  | 0.26 | 0.09 | -0.84 | -0.88 | -0.43 | 0.24 | 0.03 | -0.83 | -0.87 | 0.45 | 0.47 | 0.52 |
| T_CASO4 | 0.44 | -0.53 | 0.53 | 0.26 |  | -0.22 | -0.16 | -0.05 | 0.15 | 0.41 | -0.15 | -0.44 | -0.31 | 0.87 | -0.13 | -0.12 |
| T_CEC_CLAY | -0.08 | 0.11 | -0.67 | 0.09 | -0.22 |  | -0.10 | -0.12 | 0.07 | -0.09 | 0.01 | 0.04 | -0.06 | -0.12 | -0.06 | -0.09 |
| T_CEC_SOIL | -0.52 | 0.73 | 0.11 | -0.84 | -0.16 | -0.10 |  | 0.75 | 0.12 | -0.60 | 0.26 | 0.86 | 0.93 | -0.25 | -0.18 | -0.23 |
| T_CLAY | -0.06 | 0.27 | 0.23 | -0.88 | -0.05 | -0.12 | 0.75 |  | 0.71 | 0.02 | -0.31 | 0.73 | 0.77 | -0.15 | -0.75 | -0.76 |
| T_ECE | 0.44 | -0.33 | 0.14 | -0.43 | 0.15 | 0.07 | 0.12 | 0.71 |  | 0.65 | -0.64 | 0.11 | 0.18 | 0.13 | -1.00 | -0.98 |
| T_ESP | 0.75 | -0.84 | 0.24 | 0.24 | 0.41 | -0.09 | -0.60 | 0.02 | 0.65 |  | -0.59 | -0.57 | -0.52 | 0.42 | -0.59 | -0.53 |
| T_GRAVEL | -0.46 | 0.56 | 0.07 | 0.03 | -0.15 | 0.01 | 0.26 | -0.31 | -0.64 | -0.59 |  | 0.07 | 0.10 | 0.01 | 0.63 | 0.64 |
| T_OC | -0.52 | 0.71 | -0.10 | -0.83 | -0.44 | 0.04 | 0.86 | 0.73 | 0.11 | -0.57 | 0.07 |  | 0.92 | -0.50 | -0.17 | -0.19 |
| T_PH_H2O | -0.52 | 0.65 | -0.11 | -0.87 | -0.31 | -0.06 | 0.93 | 0.77 | 0.18 | -0.52 | 0.10 | 0.92 |  | -0.38 | -0.24 | -0.28 |
| T_REF_BULK | 0.44 | -0.47 | 0.50 | 0.45 | 0.87 | -0.12 | -0.25 | -0.15 | 0.13 | 0.42 | 0.01 | -0.50 | -0.38 |  | -0.11 | -0.03 |
| T_SAND | -0.39 | 0.27 | -0.15 | 0.47 | -0.13 | -0.06 | -0.18 | -0.75 | -1.00 | -0.59 | 0.63 | -0.17 | -0.24 | -0.11 |  | 0.99 |
| T_SILT | -0.35 | 0.24 | -0.09 | 0.52 | -0.12 | -0.09 | -0.23 | -0.76 | -0.98 | -0.53 | 0.64 | -0.19 | -0.28 | -0.03 | 0.99 |  |
